# The Gene Catalog and Comparative Analysis of Gut Microbiome of Big Cats Provide New Insights on *Panthera* Species

**DOI:** 10.3389/fmicb.2020.01012

**Published:** 2020-06-04

**Authors:** Parul Mittal, Rituja Saxena, Atul Gupta, Shruti Mahajan, Vineet K. Sharma

**Affiliations:** ^1^Metagenomics and Systems Biology Group, Department of Biological Sciences, Indian Institute of Science Education and Research Bhopal, Bhopal, India; ^2^Van Vihar National Park, Bhopal, India

**Keywords:** gut microbiome, big cats, hypercarnivores, metagenomics, Indian *Panthera*

## Abstract

Majority of metagenomic studies in the last decade have focused on revealing the gut microbiomes of humans, rodents, and ruminants; however, the gut microbiome and genic information (gene catalog) of large felids such as *Panthera* species are largely unknown to date. In this study, the gut bacterial, fungal, and viral metagenomic composition was assessed from three *Panthera* species (lion, leopard, and tiger) of Indian origin, which were consuming the same diet and belonged to the same geographical location. A non-redundant bacterial gene catalog of the *Panthera* gut consisting of 1,507,035 putative genes was constructed from 27 *Panthera* individuals, which revealed a higher abundance of purine metabolism genes correlating with their purine-rich dietary intake. Analysis with Carbohydrate Active enZyme (CAZy) and MEROPS databases identified enrichment of glycoside hydrolases (GHs), glycoside-transferases, and collagenases in the gut, which are important for nutrient acquisition from animal biomass. The bacterial, fungal, and viral community analysis provided the first comprehensive insights into the *Panthera*-specific microbial community. The *Panthera* gene catalog and the largest comparative study of the gut bacterial composition of 68 individuals of Carnivora species from different geographical locations and diet underscore the role of diet and geography in shaping the *Panthera* gut microbiome, which is significant for the health and conservation management of these highly endangered species.

## Introduction

The mammalian gut is colonized by a community of diverse microorganisms that are essential for the maintenance of host health, metabolism, and homeostasis (Ley et al., 2008b; Mcfall-Ngai et al., 2013; Mandal et al., 2015). The gut microbial community is affected by several factors, among which diet is the most crucial factor in shaping it (Ley et al., 2008a; David et al., 2014). The gut microbiome composition is well-studied for humans, rodents, and ruminants; however, its composition in the felids is largely unexplored (Ley et al., 2008b; Qin et al., 2010). A few previous studies have indicated that the gut community in felids is composed of several hundred microbial phylotypes with predominance of phylum Firmicutes followed by the phyla Proteobacteria, Actinobacteria, and Bacteroidetes (Barry et al., 2012; Tun et al., 2012; Menke et al., 2014; Duarte et al., 2016; He et al., 2018a; Zhu et al., 2018). At the genus level, *Fusobacterium*, *Clostridium*, *Sutterella*, *Fusibacter*, *Collinsella*, *Escherichia*, *Peptostreptococcus*, and *Phascolarctobacterium* have been found abundant in the felids (Barry et al., 2012; Tun et al., 2012; Menke et al., 2014; Duarte et al., 2016; He et al., 2018a; Zhu et al., 2018). The felids gut microbiome is specialized to use proteins as an energy source and is affected by the amount of fiber content in the diet (Barry et al., 2012; Di Sabatino, 2019).

Among the felids, species of the genus *Panthera* are the largest felids that reside at the top of the food chain and are also among the highly endangered species in the world. They are obligate carnivores, adapted to consume high-protein and fat diets, which is substantially different from the diet of other mammals. Recently, enormous efforts have been made to reveal the genomic basis of evolution and adaptation to hypercarnivory in these species (Cho et al., 2013; Kim et al., 2016; Figueiro et al., 2017; Mittal et al., 2019). However, understanding of the vast genetic repertoire of the gut microbiome is also essential to elucidate their adaptations. It is also likely to help in studying the gut microbiome–host metabolism interactions, adaptation to hypercarnivory, and for devising better health, conservation, and population management strategies of these highly endangered species (Bahrndorff et al., 2016).

To elucidate the microbial composition and functional repertoire, metagenomic analysis of 21 individuals of three *Panthera* species, lion (*Panthera leo*), leopard (*Panthera pardus*), and tiger (*Panthera tigris*), from Central India was carried out in this study. The individuals from these three species were on the same diet and location, which makes it a unique study to assess the influence of genetic factors in shaping the gut microbiome, while keeping diet and location constant. The structure of the gut bacterial community was determined using 16S rRNA amplicon and shotgun metagenomic sequencing. The fungal community was assessed using ITS1 region amplicon sequencing, and sequencing of virus-like particles (VLPs) revealed the viral community structure in the gut. A comparative analysis of gut microbial composition and functional profiles of *Panthera* species with other species of order Carnivora provided novel insights into the gut microbiome of these species.

## Materials and Methods

### Sample Collection and Metagenomic DNA Extraction

Fecal samples of healthy lion (*n* = 3), Indian leopard (*n* = 9), and Bengal tiger (*n* = 9) were collected fresh in nature from the Van Vihar National Park, Bhopal, India, and brought to the laboratory at 4°C immediately after collection. Bacterial metagenomic DNA was isolated from all the fecal samples using QIAamp Stool Mini Kit (Qiagen, Valencia, CA, United States) according to the manufacturer’s instructions.

The fungal DNA was extracted by using Mo Bio Power Soil DNA isolation kit (Mo Bio Laboratories, United States) with minor modifications. Approximately 200 mg of each fecal sample was added to the PowerBead tubes with 200 μl zymolyase 20 T solution (0.01% in 0.1 M sorbitol) (MP Biomedicals, Irvine, CA, United States). The tubes with samples were vortexed for a few seconds and incubated at 30°C for 30 min. After addition of 60 μl of Solution C1, the tubes were allowed to bead-beat at 4,600 r/min for 280 s. The tubes were then centrifuged at 10,000 × *g* for 1 min. Further steps were followed as per the manufacturer’s instructions. The DNA was eluted in 50 μl of elution buffer (Qiagen A, United States). The extracted fungal DNA was used for amplification of fungal ITS1 region.

Fungal and bacterial DNA concentration was estimated by Qubit HS dsDNA assay kit (Invitrogen, Carlsbad, CA, United States), and quality was estimated by agarose gel electrophoresis. All the DNA samples were stored at −80°C until sequencing.

### Bacterial 16S rRNA V3 and Fungal ITS1 Amplification

Equal concentration of bacterial and fungal DNA (∼1 ng) was used for PCR amplification of bacterial 16S rRNA V3 hypervariable region and fungal ITS1 region, respectively. The amplification of bacterial 16S rRNA V3 region was performed using Illumina Nextera XT adapter-ligated eubacterial V3 region-specific primers, 341F and 534R, with three different base modifications (Wang and Qian, 2009; Soergel et al., 2012). Nucleotide bases were introduced in different numbers to increase the overall sequence diversity of the samples, thus improving the quality of the sequenced data. Bacterial DNA samples were divided into three groups and amplified using the three different primers. However, since ITS1 sequences are quite diverse across the fungal species (Wang et al., 2015), this approach was not used for fungal ITS1 amplification.

Primer sequences for amplification of bacterial 16S rRNA V3 region are as below (the base inclusions are marked in bold): The underlined regions in all the primer sequences are the Illumina Nextera XT adapter overhangs, whereas the non-underlined regions are the primer sequences known to target eubacterial 16S rRNA V3 region or fungal ITS1 region, respectively.

1. 341F_ADA_2B
  5′ TCGTCGGCAGCGTCAGATGTGTATAAGAGACAG **CT**CCTACGGGAGGCAGCAG 3′
  534R_ADA_2B
  5′ GTCTCGTGGGCTCGGAGATGTGTATAAGAGACAG **CT**ATTACCGCGGCTGCTGGC 3′
2. 341F_ADA_3B
  5′ TCGTCGGCAGCGTCAGATGTGTATAAGAGACAG **CAT**CCTACGGGAGGCAGCAG 3′
  534R_ADA_3B
  5′ GTCTCGTGGGCTCGGAGATGTGTATAAGAGAC AG**ACT**ATTACCGCGGCTGCTGGC 3′
3. 341F_ADA_4B
  5′ TCGTCGGCAGCGTCAGATGTGTATAAGAGACAG **TCAT**CCTACGGGAGGCAGCAG 3′
  534R_ADA_4B
  5′ GTCTCGTGGGCTCGGAGATGTGTATAAGAGAC AG**CTAT**ATTACCGCGGCTGCTGGC 3′.

The optimized PCR conditions were: initial denaturation at 94°C for 5 min, followed by 35 cycles of denaturation at 94°C for 30 s, annealing at 69°C for 30 s, extension at 72°C for 30 s, and a final extension cycle at 72°C for 5 min. Paq5000 DNA polymerase (Agilent Technologies, United States) was used, and 5% dimethyl sulfoxide (DMSO) was added to the master mix to enhance the concentration of amplified product from the metagenomic template.

The amplification of fungal ITS1 region was performed using Illumina Nextera XT adapter-ligated ITS1 region-specific primers, ITS1-ADA-F and ITS1-ADA-R (Ihrmark et al., 2012; Tonge et al., 2014).

Primer sequences for amplification of fungal ITS1 region:

ITS1-ADA-F

5′ TCGTCGGCAGCGTCAGATGTGTATAAGAGACAGCT TGGTCATTTAGAGGAAGTAA 3′

ITS1-ADA-R

5′ GTCTCGTGGGCTCGGAGATGTGTATAAGAGACAGG CTGCGTTCTTCATCGATGC 3′.

The optimized PCR conditions were: initial denaturation at 95°C for 15 min (polymerase was added after this stage), followed by 35 cycles of denaturation at 94°C for 1 min, annealing at 66.5°C for 2 min, extension at 72°C for 2 min, and a final extension cycle at 72°C for 10 min. Paq5000 DNA polymerase (Agilent Technologies, United States) was used, and a final concentration of 2.5 mM MgCl_2_ was added to the PCR master mix to enhance the amplification of ITS1 region.

### Extraction of Virus-Like Particles for Virome Analysis

The purification of VLPs and DNA extraction was carried out by minor modification in the protocol suggested by Reyes et al. (2015). Approximately 100 mg of animal fecal sample was suspended in 800 μl of SM buffer [100 mM NaCl, 8 mM MgSO4, 50 mM Tris (pH 7.5), and 0.002% gelatin (wt/vol)] and homogenized by vortexing for 5 min. For removal of large particles and bacterial cells, the tubes were centrifuged twice at 5,000 × *g* for 10 min at 4°C. The supernatant was passed once through 0.45-μm pore diameter syringe filter and twice through 0.22-μm pore diameter syringe filter. Then, 20 μl lysozyme (10 mg/ml) was added to the filtrate and incubated at 37°C for 30 min, followed by 10 min incubation with 0.2 volume of chloroform. The samples were then centrifuged at 2,500 × *g* for 5 min at room temperature. The aqueous phase was collected and incubated with 3 U of DNase-I and 20 μl of 10 × DNase buffer for 1 h at 37°C. Further, the inactivation of DNase-I was done by incubating the samples at 65°C for 15 min. The samples were then incubated with 10 μl of 10% sodium dodecyl sulfate (SDS) and 1 μl of 20 mg/ml proteinase K for 20 min at 56°C, and further incubation for 10 min at 65°C with 35 μl of 5 M NaCl and 28 μl of cetyl trimethylammonium bromide solution (10% CTAB in 0.7 M NaCl). Equal volume of phenol:chloroform:isoamyl alcohol (25:24:1) was added and centrifuged at 8,000 × *g* for 5 min at room temperature. The aqueous phase was collected and centrifuged at 8,000 × *g* for 5 min at room temperature with an equal volume of chloroform.

The aqueous phase mixed with 500 μl ethanol was passed through DNeasy Mini spin column (Qiagen, United States) at ≥6,000 × *g* for 1 min. The remaining steps for elution were performed using the DNeasy Blood and Tissue Kit (Qiagen, United States) according to the manufacturer’s protocol, and finally the DNA was eluted in 20 μl of elution buffer. The extracted DNA was amplified using Illustra GenomiPhi V2 DNA amplification kit, according to the manufacturer’s instructions. The DNA was purified and eluted in 20 μl of elution buffer using DNeasy Blood and tissue kit’s elution protocol. Quality control assays were performed using 16S rDNA PCR to confirm the absent to negligible bacterial DNA contamination using the above protocol.

### Bacterial 16S rRNA V3 and Fungal ITS1 Sequencing

After evaluating the amplified products on 2% w/v agarose gel, the products were purified using Ampure XP kit (Beckman Coulter, Brea, CA, United States). The libraries were prepared using Illumina 16S metagenomic library preparation guide and evaluated on 2100 Bioanalyzer using Bioanalyzer DNA 1000 kit (Agilent, United States) to estimate the library size and Qubit 2.0 flourometer using Qubit dsDNA HS kit (Life technologies, United States) to estimate the library concentration. After this, 150 bp paired-end sequencing of both the libraries was performed on the Illumina NextSeq 500 platform (Illumina, United States) using NextSeq 500/550 v2 sequencing reagent kit (Illumina Inc., United States) at the Next-Generation Sequencing (NGS) Facility, Indian Institute of Science Education and Research (IISER) Bhopal, India.

### Shotgun Metagenome and Virome Sequencing

The extracted metagenomic and virome DNA were used to prepare the sequencing libraries using Illumina Nextera XT sample preparation kit (Illumina Inc., United States) by following the manufacturer’s protocol. Size of all the libraries was assessed on the Agilent 2100 Bioanalyzer using Agilent high sensitivity DNA kit (Agilent Technologies, Santa Clara, CA, United States) and were quantified on a Qubit 2.0 fluorometer using Qubit dsDNA HS kit (Life Technologies, United States) and by qPCR using KAPA SYBR FAST qPCR Master mix and Illumina standards and primer premix (KAPA Biosystems, Wilmington, MA, United States) following the Illumina-suggested protocol. Both the shotgun metagenomic and virome libraries were loaded on Illumina NextSeq 500 platform using NextSeq 500/550 v2 sequencing reagent kit (Illumina Inc., United States), and 150 bp paired-end sequencing was performed at the NGS Facility, IISER Bhopal, India.

### 16S and ITS-1 Amplicon Analysis

Raw sequencing reads were processed to obtain high-quality reads for further analysis. First, the ambiguous bases in the 5′ or 3′ ends of the raw reads were trimmed using NGSQC toolkit (v2.3.3) (Patel and Jain, 2012), and then reads with >3 ambiguous bases and length <60 bases were removed. Paired-end reads were merged to single long reads using FLASH (v1.2.11) (Magoc and Salzberg, 2011), and the reads having quality more than 30 for 80% bases were considered as high-quality reads for further analysis. The primer sequences from these reads were trimmed from both ends using cutadapt (v1.8.3) (Martin, 2011), and the reads without the primer sequences were discarded.

The high-quality reads were then clustered at 97% identity against Greengenes database (v13_8) as reference using QIIME (v1.9.1) for 16S amplicons (Caporaso et al., 2010). The ntf ITS-1 database was used as a reference database for taxonomic assignment (Motooka et al., 2017). For the reads which failed closed reference picking, *de novo* clustering was performed at 97% identity using UCLUST and they were assigned using lowest common ancestor approach against the Greengenes database for 16S and ntf ITS-1 database for ITS1 amplicons. The operational taxonomic units (OTUs) with less than 100 sequences were filtered out from the analysis.

### Comparative Analysis

The bacterial community structure of nine Bengal tigers, nine Indian leopards, and three lions used in this study were compared with other datasets obtained from NCBI SRA: 13 North Chinese leopards and eight Amur leopards (SRR7229827-47), two Malayan tigers (SRR6147361 and SRR6147362), one caracal (SRR6841704), three cheetahs (ERR2821256, ERR2821310, and ERR2821311), five African wild dogs (ERR2821271, ERR2821340-43), four wolves (ERR2821250-51, ERR2821276, ERR2821345), and five foxes (ERR1804913-17). The raw reads were processed to obtain high-quality reads for all samples as mentioned above. Data produced using Ion torrent were first converted to Illumina format using NGSQC toolkit and then processed further. For single-end reads, the step involving merging using FLASH was skipped. Closed reference followed by *de novo* clustering of remaining reads was performed using similar criteria mentioned above.

The alpha diversity of bacteria in each sample was assessed using Shannon indices and observed OTUs, and rarefactions were performed from 100 sequences to a maximum of 560,000 sequences using QIIME. The beta diversity was estimated using weighted UniFrac distances in QIIME. The alpha diversity of fungi in each sample was assessed using Shannon indices and observed OTUs, and rarefactions were performed from 100 sequences to a maximum of 10,000 sequences using QIIME. The principal component analysis (PCA) of taxonomic profiles of all samples was performed using PCA–sklearn in Python. The genus with >1% abundance in either sample was considered in this analysis. The top two principal components were plotted.

### Virome Analysis

Raw metagenomic reads of seven Bengal tigers, nine Indian leopards, and three lions generated in this study were processed to obtain high-quality reads for further analysis. Firstly, the low-quality bases in the 5′ or 3′ ends were removed from the raw reads, and then ambiguous bases at both ends were trimmed using NGSQC toolkit (v2.3.3) (Patel and Jain, 2012). The reads having quality more than 30 for 80% bases were considered as high-quality reads for further analysis. The filtered reads were aligned against a downloaded set of 2,401 bacterial genomes from NCBI RefSeq to remove bacterial contamination in the reads. Since virus genomes usually carry 10% of bacterial genome, a coverage-based criterion was used to identify the bacterial contaminants as described previously (Moreno-Gallego et al., 2019) using Bowtie-2 (v2.2.3) for alignment and Samtools (v1.4) for calculating the bacterial coverage. The reads mapping to these bacterial contaminants were discarded. The remaining reads were used for *de novo* assembly using metaSPAdes (Nurk et al., 2017) for each sample separately. The identified contigs and reads from each sample were pooled, and a second round of assembly was performed using metaSPAdes (Nurk et al., 2017). The reads were then mapped to these contigs for quantification.

A total of 11,345 viral genomes were downloaded from NCBI along with their taxonomic information. The taxonomic assignment of contigs at family level was performed using dc_megablast against the downloaded viral genomes as described previously (Norman et al., 2015).

### Correlation of Bacterial, Fungal, and Viral Taxa

To examine the correlations among bacterial, fungal, and viral taxa, pair-wise Spearman correlation was calculated between (1) bacterial and fungal genera, (2) fungal genera and viral families, and (3) bacterial genera and viral families in R. The taxa with at least 1% abundance in mean values were used to find the correlations.

### Shotgun Metagenomic Analysis

Raw metagenomic reads of nine Bengal tigers, nine Indian leopards, and three lions generated in this study were processed to obtain high-quality reads for further analysis. Sequencing reads from six Amur tigers were also obtained from NCBI SRA database (SRR6155883-85, SRR6256454-56). First, the low-quality bases in the 5′ or 3′ ends were removed from the raw reads, and then ambiguous bases at both ends were trimmed using NGSQC toolkit (v2.3.3) (Patel and Jain, 2012). The reads having quality more than 30 for 80% bases were considered as high-quality reads for further analysis. The filtered reads were aligned with mammalian genomes to remove human/host contamination in the reads.

#### Construction of Gene Catalog

*De novo* assembly of all the samples was performed using high-quality reads with metaSPAdes (v3.13.0) (Bankevich et al., 2012). Genes were predicted on the contigs (>300 length), obtained after assembly, using MetaGeneMark (Zhu et al., 2010). The reads were also aligned to microbial genomes obtained from NCBI RefSeq and HMP to identify highly abundant species using metagenomic data. The alignment was performed using blastn with cutoff parameters: identity ≥ 90% and e-value < 1e-06 in the top hit. The complete gene sets of microbes were retrieved when more than 5% of the reads in any sample mapped to the microbial genome. Thus, the gene sets of 33 microbial species were retrieved from NCBI RefSeq. To construct a non-redundant gene catalog, the genes obtained after prediction from contigs and from the top abundant microbial species were clustered using CD-HIT (v4.6) (Li and Godzik, 2006) using a sequence identity cutoff of 0.95 and minimum coverage cutoff of 0.9 for shorter sequences as reported previously (Karlsson et al., 2013).

#### Relative Gene Abundance and Functional Annotation

To assess the abundance of genes, reads were aligned to the gene catalog using Bowtie2 (v2.2.3) (Langmead and Salzberg, 2012). For each primary alignment, the mapped read was counted as one copy. The copy number of each gene and its relative abundance were calculated as described previously (Qin et al., 2012).

The putative amino acid sequences which translated from the gene catalog were aligned against the Kyoto Encyclopedia of Genes and Genomes (KEGG), Carbohydrate Active enZyme (CAZy) (Lombard et al., 2014), and MEROPS (Rawlings et al., 2014) using blastp (e-value < 1e-5), and each protein was assigned by the highest scoring hit(s) containing at least one HSP scoring over 60 bits and identity >60.

## Results

In this study, a comprehensive analysis of the gut metagenomic community of *Panthera* species was carried out to study the bacterial, fungal, and viral community structure. A total of 21 individuals from three *Panthera* species, lion, leopard, and tiger, were included in this study (Supplementary Table 1). All the individuals were on the same carnivorous diet at the same location, which removes any bias arising due to varying diet and geography.

### Metagenomic Diversity of the *Panthera* Gut

**FIGURE 1:**
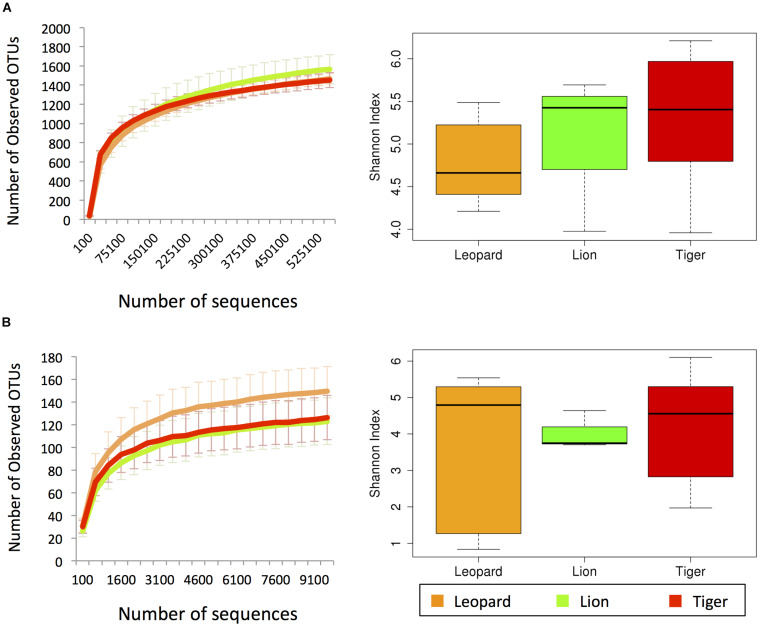
The alpha diversity estimates from **(A)** V3 hypervariable region of 16S rRNA and **(B)** ITS-1 amplicon sequences. The left charts show the rarefaction curves of the number of observed operational taxonomic units (OTUs) with respect to the number of sequences. The box plots in the right show the Shannon index estimates using the maximum number of sequences.

The bacterial diversity of 21 individuals from three *Panthera* species was estimated using 27,032,912 paired-end reads (∼1.3 million reads per sample) generated from amplicon sequencing of V3 hypervariable region of 16S rRNA gene (Supplementary Table 2). The fungal diversity was estimated using 12,191,230 paired-end reads (∼0.76 million reads per sample) of ITS1 region from 16 (out of 21) individuals. A total of 2,727 bacterial OTUs were identified in the samples after clustering at 97% identity, among which 1,785 OTUs were obtained by using reference-based clustering, and 942 OTUs were obtained by *de novo* clustering. A total of 392 OTUs were identified after clustering the ITS1 amplicon sequences. The rarefactions analysis revealed that the amount of sequences generated per sample sufficiently estimates the bacterial and fungal species diversity (Figure 1). The Shannon diversity index of bacterial community varied in the range of 3.9–6.2, which is similar to other free-ranging carnivore species (Menke et al., 2014). The fungal diversity showed substantial variation among the individuals, ranging from 0.8 to 6.1 (Supplementary Figure 1).

#### Phylogenetic Assessment of Bacterial Gut Microbiome of *Panthera*

**FIGURE 2:**
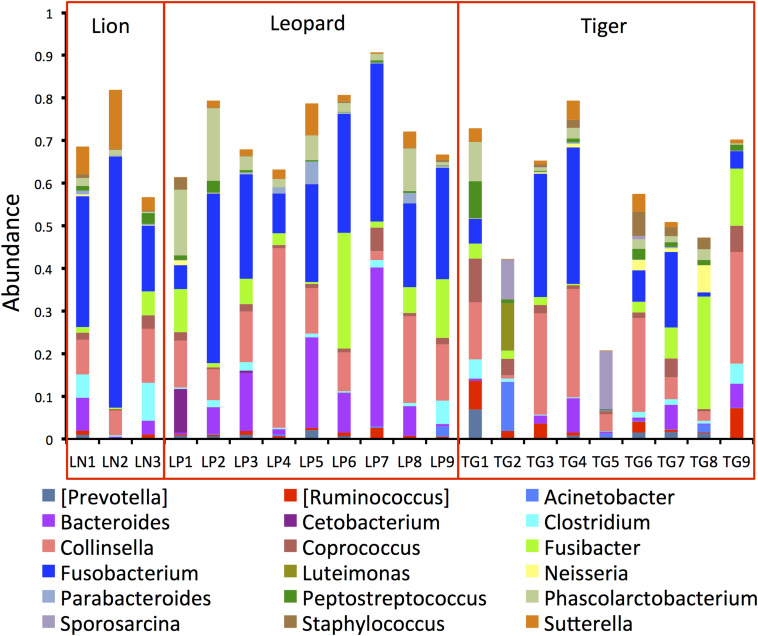
Bar plots showing the topmost abundant bacterial genera present in the *Panthera* gut microbiome. The genera with more than 1% abundance are shown.

The phylogenetic assignment of the bacterial OTUs revealed that the phylum Fusobacteria was the most abundant phylum in lion (35%), and phylum Firmicutes was most abundant in tiger (40%) and leopard (32%) (Supplementary Figure 2). These phyla were also found abundant in the earlier feline gut microbiome studies, with Firmicutes showing the highest abundance (Ritchie et al., 2008; Desai et al., 2009; Handl et al., 2011; Delsuc et al., 2014; He et al., 2018b). At the genus level, *Fusobacterium* showed the highest abundance in lion (35%) and leopard (24%) samples, and the genus *Collinsella* was the most abundant in tiger (14%) (Figure 2). A recent study on Amur tigers showed a higher abundance of *Escherichia* (24%) in their gut microbiome; however, in this study, its abundance was less than 1% in all the *Panthera* individuals (He et al., 2018a). Other bacterial genera, including *Sutterella*, *Clostridium*, *Fusibacter*, *Peptostreptococcus*, and *Phascolarctobacterium*, showed abundance between 1 and 7% in the *Panthera* gut. These genera have also been identified as abundant in tiger, cheetah, and jackal previously (Menke et al., 2014; He et al., 2018a, b). A large number of sequences (22%) could not be assigned at the genus level, and their count showed substantial variation among the samples (5–64%). The highest number of unassigned sequences was identified in one individual of tiger, TG5 in which 45% of reads were assigned to an unknown genus of family *Pseudomonadaceae*. Significant differences in the abundance of bacterial genera *Bacteroides* and *Parabacteroides* between leopard and tiger and *Sutterella* between lion and tiger were observed (Mann–Whitney *U*-test, *P*-value < 0.05; Supplementary Figure 3). This suggests that species-specific differences exist in the gut microbiome even in the closely related *Panthera* species having a similar diet.

#### Phylogenetic Assessment of *Panthera* Gut Mycobiome

**FIGURE 3:**
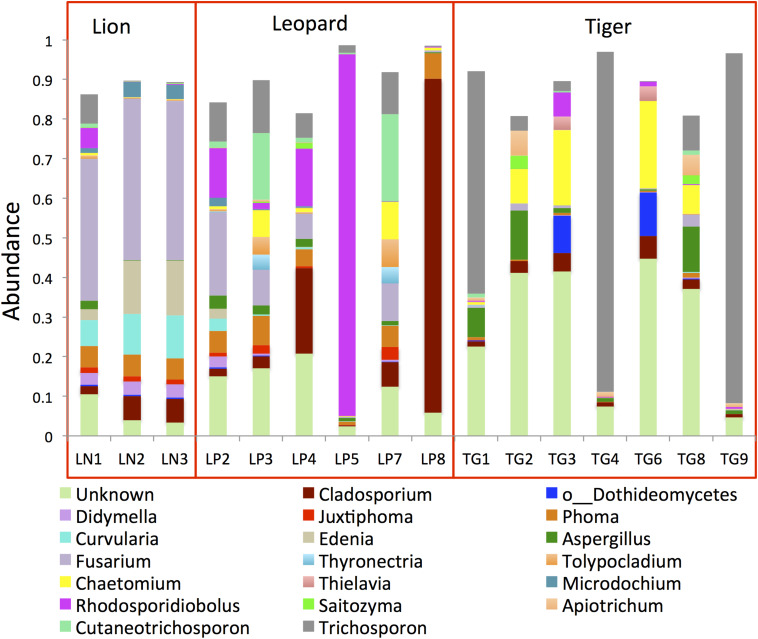
Bar plots showing the topmost abundant fungal genera present in the *Panthera* gut microbiome. The genera with more than 1% abundance are shown.

Analysis of ITS1 OTUs revealed the predominance of Ascomycota (50%) and Basidiomycota (32%) in the gut (Supplementary Figure 4). Two other phyla, Mucoromycota and Olpidiomycota, were detected in smaller proportions (<0.2%) in few samples. The proportions of the topmost abundant fungal genera showed drastic variations among the samples (Figure 3). The genus *Trichosporon* (mean abundance: 18%) was identified as the most abundant (>80%) genus in a few samples and less than 10% in most samples of the *Panthera* species. The genus *Fusarium* (10%) was found as the second most abundant genus in the *Panthera* gut. Other genera, including *Cladosporium*, *Rhodosporidiobolus*, *Chaetomium*, *Phoma*, *Aspergillus*, *Cutaneotrichosporon*, and *Edenia*, were also found abundant (>2%) in the gut. Species of genus *Trichosporon* and *Aspergillus* have been found abundant in felids.

#### Phylogenetic Assessment of *Panthera* Gut Virome

**FIGURE 4:**
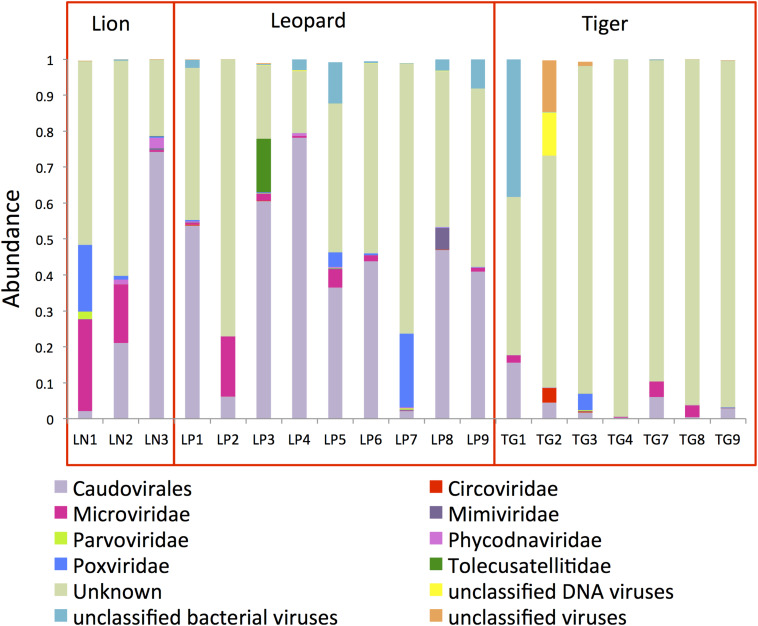
Bar plots showing the topmost abundant virus families present in the *Panthera* gut microbiome. The families with more than 1% abundance are shown.

A total of 31,446,701 paired-ends reads were generated after sequencing of the VLPs. Taxonomic assignment of the virome contigs majorly identified *Caudovirales*, *Poxviridae*, *Mimiviridae*, and *Microviridae* family of viruses. Only 13% of virome contigs could be assigned to a taxonomic level, indicating the enormous sequence variation present in these viruses. The viral genomic reads assignment and quantification revealed a higher abundance of *Caudovirales* viruses in the *Panthera* gut (32% in lion and 41% in leopard) (Figure 4). *Microviridae* (14%) and *Poxviridae* (6%) also showed high abundance in lion individuals. In tiger, 82% of the reads could not be assigned to any known viruses. Previous studies suggest that viruses belonging to *Caudovirales* and *Microviridae* are commonly found in the mammalian gut microbiome (Mills et al., 2013; Xu et al., 2013; Di Sabatino, 2019). Phage such as Twort from the order *Caudovirales* has been identified as abundant in the feline and canine gut microbiota previously (Tun et al., 2012; Di Sabatino, 2019). In this study, *Clostridium* phages “phiS63,” “c-st,” “phi24R,” “phi8074-B1,” and *Bacteroides* phage “B40-8” from the order *Caudovirales* were among the highly abundant viruses in the *Panthera* feces (Supplementary Data Sheet 1). A large number of *Clostridium* phages (282 contigs) and their higher abundance were identified in the *Panthera* feces. In *Microviridae* family, “Lynx canadesis associated microvirus CLP 9413,” previously identified in feline feces (Kraberger et al., 2018), was among the most abundant viruses in the *Panthera* gut. “BeAn 58058” virus from *Poxviridae* family was also found abundant in the *Panthera* gut. The Poxvirus “BeAn 58058,” first isolated from a rodent species in Brazil, was the only eukaryotic virus detected in this study.

#### Correlation of Bacterial, Fungal, and Viral Taxa

Pair-wise Spearman correlations were calculated among the bacterial genera, fungal genera, and viral families. The analysis revealed a significant negative correlation of *Collinsella* and *Fusarium* (ρ = −0.5), *Ruminococcus* with *Phoma* and *Culvularia* (ρ = −0.5), and *Sutrella* with *Aspergillus* and *Chaetomium* (ρ = −0.6) (Supplementary Table 3). Positive correlations of *Caudovirales* and fungal genera *Phoma* (ρ = 0.6) and *Microviridae* with *Curvularia* and *Edenia* (ρ = 0.6) were also observed.

### Functional Assessment of the *Panthera* Gut Microbiome

Functional analysis of the gut microbiome was carried out using 552,953,078 high-quality metagenomic reads from 27 *Panthera* individuals (Supplementary Table 4). These included data from 21 individuals from this study and data from six Amur tiger individuals obtained from previous studies (He et al., 2018a). A total of 1,507,035 open reading frames (ORFs) were identified in the gene catalog of gut metagenome after clustering the predicted ORFs. The identified genes were annotated using the KEGG database, and the genes were categorized based on KEGG Orthologs (KO) and Pathways. A total of 967,179 ORFs from the gene catalog could be assigned using the KEGG database, which corresponds to 64% of the total genes. Among the annotated genes, a total of 23,926 genes were assigned to peptidases/proteinases, and 12,800 genes were assigned to ABC transporters. Genes encoding hemolysin proteins, such as hemolysin A (hlyA), hemolysin D (hltD, cyaD), hemolysin III (hylIII), hemolysin erythrocyte lysis protein, and hemolysin activation/secretion (shlB, hhdB, hpmB), were also identified in the gene catalog of *Panthera* species gut. Perfringolysin O regulator protein, which is a cholesterol-binding cytolysin, was also identified in the gene catalog. These genes could play essential roles in the uptake of nutrients from blood and adipose tissues of animals.

**FIGURE 5:**
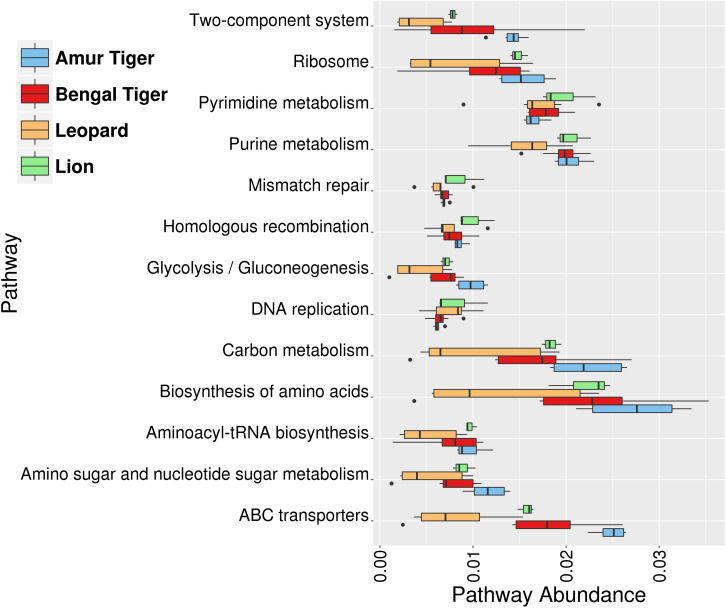
Box plots showing the top 10 most abundant functional pathways identified using the Kyoto Encyclopedia of Genes and Genomes (KEGG) annotation in gut (bacterial) microbiome of lion (green), leopard (orange), Bengal tiger (red), and Amur tiger (blue).

At the functional level, the most abundant pathways identified were “Biosynthesis of amino acids,” “Purine metabolism,” “Pyrimidine metabolism,” “Carbon metabolism,” “ABC transporters,” and “Ribosome” (Figure 5). The higher abundance of these pathways was also reported earlier in the gut microbiome of Amur tigers (He et al., 2018a). “Ribonucleoside-diphosphate reductase alpha chain” (EC1.17.4.1), “Ribonucleoside-triphosphase reductase” (EC:1.17.4.2), and dUTP pyrophosphatase (EC:3.6.1.23) were among the top 10 most abundant KOs in the gut microbiota. These genes are involved in purine and pyrimidine metabolism. Purine metabolism is essential for uric acid degradation in the intestine, and its higher abundance corroborates with previous reports on association of the pathway with purine-rich diet in these hypercarnivores (Zhu et al., 2018). The higher abundance of pathways related to genetic material processing “Mismatch repair,” “Homologous recombination,” “DNA replication,” and “Aminoacyl-tRNA biosynthesis” were also observed among the top 10 most abundant pathways.

Annotation of genes using the CAZy database revealed the higher abundance of three families of enzymes, glycosyltransferase (GT), glycoside hydrolase (GH), and carbohydrate-binding modules (CBMs). Among the GTs, the subfamilies GT2 and GT4 were the two topmost abundant subfamilies identified in *Panthera* (Supplementary Table 5). The lysozymal “active” enzymes (EC 3.2.1.17) were highly abundant in the *Panthera* gut that are possibly required for the digestion of glycerol, sugars, and amino acids from glycoproteins, glyco(amino)lipids, glyco(amino)glycans, and nucleoside diphosphate sugars. Alpha-amylase was among the other abundant carbohydrate-active enzymes, which plays vital roles in the utilization of dietary starch and proteins in mammals (Pusztai et al., 1995). A higher abundance of β-glucosidase and β-xylosidase enzymes was also observed in the gut metagenome of these species.

MEROPS is a comprehensive database of peptidases and proteinases, which are grouped into different families (Rawlings et al., 2014). Annotation of genes was carried out with MEROPS database to identify peptidases/proteinases encoded by microbes in the *Panthera* gut. The analysis identified a total of 24,526 unique MEROPS genes. These genes were highly enriched (>8% abundance) for Subfamily M23B unassigned peptidases, which are zinc metalloendopeptidases. Among other abundant enzymes were prolinases, carboxydipeptidases, and carboxypeptidases (Supplementary Table 6). High abundance of collagenases, which helps in the degradation of collagen, was also observed. Serine and cysteine peptidases were particularly abundant in the gut, which has been previously reported in the mammalian gut (Biancheri et al., 2013). The high abundance of cysteine protease ATG4 (EC:3.4.22.-) was also observed in the KEGG analysis. The identification of a large number of peptidases, including the high abundance of collagenases, in the gut microbiome correlates with the animal-based diet of these carnivores.

In addition, comparison of functional profiles of leopard and tiger gut has revealed differences in several KO (Supplementary Data Sheet 2). At the pathway level, significant differences were observed in the abundance of “Naphthalene degradation,” “ABC transporters,” “Limonene and pinene degradation,” “Xylene degradation,” “Chloroalkane and chloroalkene degradation,” and “Glycerolipid metabolism” between leopard and tiger (Mann–Whitney *U*-test, *P*-value < 0.001; Supplementary Data Sheet 3).

### Comparative Analysis of Gut Microbiome in Carnivora Species

**FIGURE 6:**
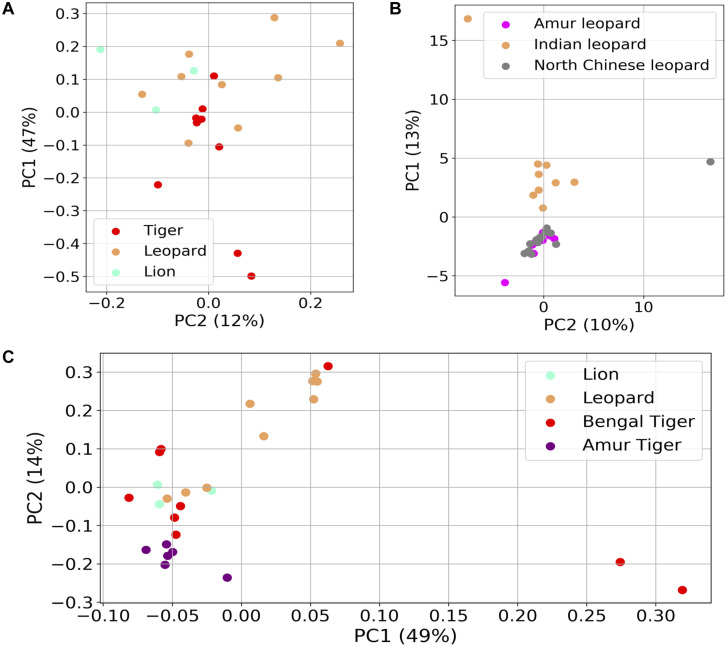
Differences in the fecal microbial communities of *Panthera* species using dimensionality reduction methods. **(A)** Principal coordinates analysis (PCoA) plot of weighted UniFrac distances of gut bacteria of the 21 individuals. **(B)** Principal component analysis (PCA) plot of gut bacterial composition of Indian leopard (this study), Amur, and North Chinese leopards (data from previous studies). **(C)** PCoA plot of Hellinger distances calculated using functional profile [Kyoto Encyclopedia of Genes and Genomes (KEGG) Orthologs] in the 21 *Panthera* individuals (this study) and six Amur tigers.

Several studies suggest that geography and diet are the critical confounding factors that can alter the gut microbiota of mammals (Fallani et al., 2010; Scott et al., 2013; David et al., 2014). The effects of these factors are minimized in this study as the subjects were living at the same geographical location and consumed the same diet. Thus, the study provides an excellent opportunity to understand the species-specific differences in bacterial diversity while the effect of two critical confounding factors was minimal. Interestingly, the principal coordinates analysis (PCoA) carried out using the weighted UniFrac distances did not show any species-specific clustering (Figure 6A). The gut bacteria showed no grouping based on host (Anosim *R*-value = 0.076, *p*-value = 0.19).

To understand whether geography plays a role in shaping the gut microbiota of these species, PCA was carried out between nine Indian leopards of this study, and eight Amur leopards and 13 North Chinese leopards from previous studies (Han et al., 2019). The analysis revealed separate clustering of Indian leopards from the two other leopard subspecies (Figure 6B). A similar observation was apparent on comparison of the functional profiles of *Panthera* gut including the Bengal and Amur tigers, where the Amur tigers were separated from the other individuals on PC1 (Figure 6C; Adonis R-squared value = 0.205, *p*-value < 0.001). The observed differences in the gut metagenome of the same *Panthera* species belonging to different geographical locations reaffirm the key impact of geographical locations in shaping the gut microbiome composition (Fallani et al., 2010).

**FIGURE 7:**
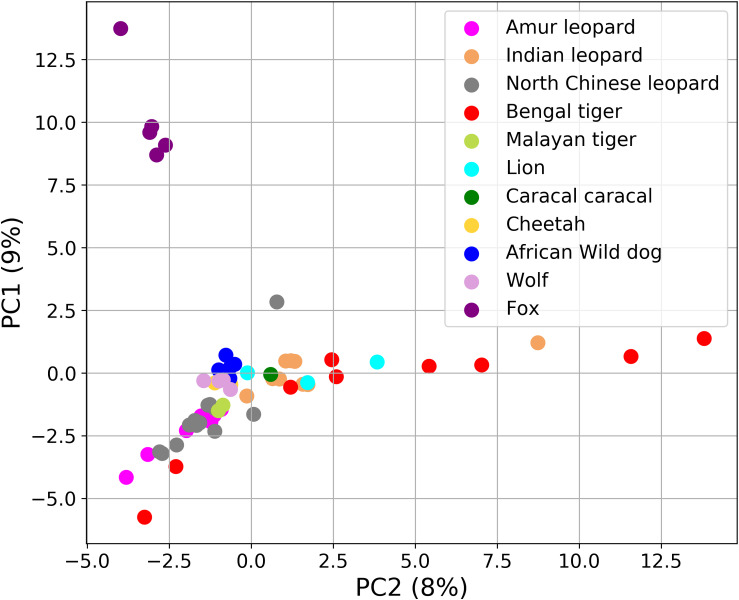
Principal component analysis (PCA) plot showing the differences in the fecal microbial communities of 62 Carnivora individuals explaining 17% of the total variance. Fox (violet color) formed a separate cluster from all other species.

To further understand the gut microbiota composition of *Panthera* species in light of previously studied composition in hypercarnivores, we analyzed the data (bacterial amplicon 16S rRNA) from 48 felids and 14 canids (Supplementary Table 7). In the PCA, the foxes (among the canids) formed a completely separate cluster from all other species (Figure 7). The separate clustering of foxes could be explained by their distinct diet that includes a variety of food including fruits, whereas all other species are obligate carnivores. These findings suggest the crucial role of diet and geography in shaping the gut microbiome of these species.

## Discussion

Recent studies have revealed that the host-associated gut microbiota significantly influences the host health and, therefore, the understanding of gut microbiota of the endangered species is important for their better management and conservation (Mandal et al., 2015; Allan et al., 2018; Trevelline et al., 2019). However, the gut microbiome of highly endangered species such as the *Panthera* species is poorly characterized. Here, we performed a comprehensive gut microbiome study of these threatened *Panthera* species present in India. The gut of mammals is majorly inhabited by bacterial, fungal, and viral (bacteriophages) species. Therefore, in this work, the bacterial, fungal, and viral species present in the gut are studied to have a complete understanding of the gut microbiota of hypercarnivores, specifically the *Panthera* species of Indian origin.

In the diversity analysis, it was apparent that the number of sequences generated per sample was sufficient to estimate the complete bacterial and fungal species diversity present in the sample (Figure 1). The higher abundance of *Fusobacterium* and *Collinsella* species observed in the *Panthera* individuals in this study was also reported previously in Amur tigers (He et al., 2018a, b). *Collinsella* has been reported to play a role in altering intestinal cholesterol absorption, decreasing glycogenesis in the liver, and increasing triglyceride synthesis in humans, and its abundance reduces with the increase in fiber-rich diet (Chow et al., 2011; Gomez-Arango et al., 2018). These findings are consistent with the high abundance of *Collinsella* in *Panthera* gut, where the fiber intake is minimal. The higher abundance of *Clostridium* and *Bacteroides* bacteriophages from the order *Caudovirales* correlates with the higher abundance of these bacteria genera as noted from 16S amplicon analysis. The correlation analysis revealed the negative correlation of *Collinsella* and *Fusarium*. This observation correlates with the previous studies, which have shown that species of *Collinsella* reduces the major mycotoxin Deoxynivalenol (DON) produced by *Fusarium* and thus reduces the pathogenic potential of *Fusarium* species (Wachowska et al., 2017). Species of *Fusobacterium*, *Bacteroides*, and *Clostridium* are not inhibited by mycotoxins produced by *Fusarium* (Roig et al., 2014). Previous reports have shown that the species of bacterial genera *Fusobacterium*, *Bacteroides*, and *Clostridium* and fungal genera *Candida*, *Aspergillus*, *Fusarium*, *Cladosporium*, and *Trichosporon* coinhabit and constitute the enterotypes of the human gut microbiome (Hugon et al., 2017). Studies on oral microbiome have also found significant correlations between *Cladosporium* and *Fusobacterium* (Sharma et al., 2019), and these two genera were found to be highly abundant in our study.

To our knowledge, this is the first study that reveals the fungal diversity in the *Panthera* species gut. Felids show a higher diversity and richness of fungal species in the gut in comparison to canids. In this study, we found a relatively higher diversity of *Panthera* in comparison to cats in terms of the number of OTUs (Handl et al., 2011). The fungal diversity showed considerable variation among the individuals. Phylogenetic assessment of ITS1 OTUs revealed the predominance of Ascomycota and Basidiomycota in the gut. The higher abundance of Basidiomycota in the mammalian gut is commonly observed; however, it was previously thought to be absent in felids and thus appears to be a novel finding of this study (Handl et al., 2011; Yang et al., 2018). *Saccharomyces* previously found abundant in felids were detected in small proportions in this study (Honneffer et al., 2014). The differences observed in the abundance of bacterial and fungal species indicate that the gut microbiota of these *Panthera* shows substantially different composition from other felids.

One of the most significant outcomes of the study was the construction of gene catalog of the gut microbiome of *Panthera* species. The catalog comprised of a total of 1,507,035 (∼1.5 million) genes of which 64% could be annotated using the KEGG database. The number of genes in the gene catalog is substantially lower compared to the human gut gene catalog, which consisted of 9.9 million genes (Li et al., 2014), pig gut microbiome (∼7.7 million genes) (Xiao et al., 2016), and mouse gut bacterial catalog (∼2.6 million) (Xiao et al., 2015). However, the size of the gene catalog is comparable to the size of *Macaca fascicularis* gene catalog (∼1.9 million) (Li et al., 2018). The lower number of genes in the constructed gene catalog of the *Panthera* gut microbiome can be attributed to the poor availability of data from different regions used to build the gene catalog. Future studies with a larger dataset may help in capturing the complete microbial gene sets in the *Panthera* gut.

The functional assessment of the gut bacteria identified a large number of metabolic genes and enzymes present in the gut of *Panthera* species gut. An important observation was enrichment in the purine metabolic pathways essential for uric acid degradation in the intestine. Its higher abundance corroborates with previous reports on the association of the pathway with purine-rich diet in these hypercarnivores (Zhu et al., 2018). The carbohydrate-active enzymes identified in this study play critical roles in the utilization of dietary starch and proteins in mammals (Pusztai et al., 1995). Studies have shown that *Bacteroides* species encode large numbers of animal-CAZys, which corroborates with the identification of relatively enriched 16S sequences assigned to the genus *Bacteroides*. The higher abundance of collagenases and other peptidases identified in the gut microbiome correlates with the animal-based protein-rich diet of these carnivores. The presence of hemolysins and cholesterol-binding cytolysins further suggests their adaptations to hypercarnivorous diet.

Among the carbohydrate-active enzymes were β-glucosidase and β-xylosidase in the gut metagenome of the *Panthera* species. The identified celluloses are required for the degradation of cellulose, which is an integral part of plant biomass and is absent in animals. A study on Iberian lynx has also reported the higher abundance of these enzymes in the gut microbiota (Alcaide et al., 2012). Although the diet of these *Panthera* species mostly comprises of higher vertebrates, a few studies have reported the presence of plant biomass in the scats of leopards, lions, and tigers (Hoppe-Dominik, 1988; Cashman et al., 1992; Mukherjee and Sen Sarkar, 2013; Stein and Hayssen, 2013). Thus, these findings suggest that the gut microbiome of wild felids not only harbors gene sets for the uptake of sugars from primary animal tissues but also from the plant biomass. The plant materials are most likely derived from their prey, and the enzymes to digest these plant tissues indicate the wide metabolic capabilities of the gut microbiome. Future studies including other carnivore species may shed more light on these observations to understand the role of these genes in carnivorous gut.

This study acts as a controlled study wherein the three *Panthera* species were on a similar diet and belonged to the same geographical location. Thus, it provides an opportunity to identify the species-specific differences in the gut microbiome composition among lion, leopard, and tiger. At the functional level, the study identified significant differences in the abundance of “Naphthalene degradation,” “ABC transporters,” “Limonene and pinene degradation,” “Xylene degradation,” “Chloroalkane and chloroalkene degradation,” and “Glycerolipid metabolism” between leopard and tiger. The PCA of several Carnivora species including the leopards from two different geographical locations suggested the crucial role of diet and geography in shaping the gut microbiome composition and the influence of host genetic factors on the gut microbiome could not be observed in the *Panthera* species. This indicates that the host phylogeny influences the gut microbiome when seen at a broader level such as the order Carnivora (Ley et al., 2008a; Muegge et al., 2011); however, at the species level, these differences are not significant and the microbial community is mostly governed by diet or other factors. The crucial role of diet in modulating the animal gut microbiome is well documented. The 21 *Panthera* individuals included in this study were captive individuals and were provided a controlled diet, which is different from the diet of other individuals and could be a major factor in determining the gut microbiome composition of these individuals. Thus, their composition significantly varied from other felid species.

## Conclusion

To summarize, this is the first study that reveals the bacterial, fungal, and viral diversity of *Panthera* gut from healthy individuals of lion, leopard, and tiger. This will enable future studies on the nutrition and dietary intake of these large terrestrial animals and may be used to compare with the gut metagenomic diversity of diseased individuals. One of the significant outcomes of this study is the establishment of a comprehensive *Panthera* gut microbiome gene catalog, which will be used as a reference resource in metagenomic studies. The identification of enzymes for the metabolism of carbohydrates and proteins in the gut microbiome not only indicates the adaptations of the host to the animal-based diet of the host but also to digest the plant-based components often identified in their scats. A comparison of the metagenomic diversity and functional potential of *Panthera* species of Indian origin with other species revealed that diet and geography play a crucial role in shaping the gut microbiome of *Panthera* species and surpass the species-species differences among the *Panthera* species.

## Data Availability Statement

The raw sequence data of gut microbiome of lions, leopards, and tigers generated in this study can be accessed using NCBI SRA database with the Accession ID SRR9943707-83 under the BioProject ID PRJNA559605.

## Ethics Statement

This study was carried out in accordance with the principles of the Basel Declaration and recommendations of Institute Ethics Committee of Indian Institute of Science Education and Research (IISER) Bhopal. The protocol was approved by the Institute Ethics Committee.

## Author Contributions

AG provided the fecal samples and necessary information of the *Panthera* individuals. RS and SM performed the experimental procedures and sequencing. PM performed the computational analysis and created figures. PM and VS interpreted the results. All the authors have read and approved the final manuscript.

## Conflict of Interest

The authors declare that the research was conducted in the absence of any commercial or financial relationships that could be construed as a potential conflict of interest.
