# Supplementary material for: The Gene Catalog and Comparative Analysis of Gut Microbiome of Big Cats Provide New Insights on Panthera Species
Source: Front Microbiol. 2020 Jun 4;11:1012. doi: 10.3389/fmicb.2020.01012 (PMC7287027; doi:10.3389/fmicb.2020.01012)
Supplement: Supplementary file 4 [file Data_Sheet_4.DOCX]

**Supplementary**

**The gene catalogue and comparative analysis of gut microbiome of big cats provide new insights on *Panthera* species**

**Supplementary Tables**

**Supplemenatary Table 1.** Detailed information of the fecal samples used in this study.

| **S. No.** | **Species** | **Name** | **Sex** | **Age** | **Weight** | **Brought from** | **Date of sample collection** | **Origin** |
| --- | --- | --- | --- | --- | --- | --- | --- | --- |
| 1 | Tiger | Kanha | M | 12 Y | - | Kanha Tiger Reserve Mandla MP | 03/30/17 | Wild |
| 2 | Tiger | Bandhu | M | 04 Y | 210 kgs | Bandhavgarh Tiger Reserve Mandla MP | 03/30/17 | Wild |
| 3 | Tiger | Panna | M | 06 Y | 190 kgs | Kanha Tiger Reserve Mandla MP | 03/30/17 | Wild |
| 4 | Tiger | Gauri | M | 12 Y 09 M | 140 kgs | Jaipur Zoo | 03/30/17 | Captive |
| 5 | Tiger | Matakkali | F | 05 Y | 140 kgs | Satpura Tiger Reserve Mandla MP | 03/30/17 | Wild |
| 6 | White Tiger | Riddhi | F | 08 Y | 135 kgs | Kamla Nehru Prani Sangrahalay Indore MP | 03/30/17 | Captive |
| 7 | Tiger | Kamlesh | F | 09 Y | 130 kgs | Kamla Nehru Prani Sangrahalay Indore MP | 04/04/17 | Captive |
| 8 | Tiger | Ganga | F | 02 Y 04 M | 140 kgs | Kamla Nehru Prani Sangrahalay Indore MP | 04/04/17 | Captive |
| 9 | Tiger | Priya | F | 18 Y | 86 kgs | Jaipur | 04/04/17 | Rescue from circus |
| 10 | Hybrid Lion | Shiva | M | 18 Y | 120 kgs | Jaipur | 03/30/17 | Rescue from circus |
| 11 | Hybrid Lion | Shivani | F | 18 Y | - | Jaipur | 03/30/17 | Rescue from circus |
| 12 | Hybrid Lion | Jamuna-2 | F | 24 Y | - | - | 03/30/17 | Rescue from circus |
| 13 | Leopard | Nena | F | 25 Y | - | Neemuch Forest Division MP | 03/30/17 | Wild |
| 14 | Leopard | Puri | F | 04 Y | - | Bhamahpuri Forest Division MH | 03/30/17 | Wild |
| 15 | Leopard | Nimu | M | 20 Y | - | Neemuch Forest Division MP | 03/30/17 | Wild |
| 16 | Leopard | Panna | M | 13 Y | 60 kgs | Panna Forest Division MP | 03/30/17 | Wild |
| 17 | Leopard | Jabalpur | M | 12 Y | 60 kgs | Jabalpur Forest Division MP | 03/30/17 | Wild |
| 18 | Leopard | Jobat | M | 06 Y | 65 kgs | Kamla Nehru Prani Sangrahalay Indore MP | 04/04/17 | Wild |
| 19 | Leopard | Raju | M | 05 Y 06 M | 60 kgs | Bhamahpuri Forest Division MH | 04/04/17 | Wild |
| 20 | Leopard | Sheru | M | 07 Y 06 M | 60 kgs | Bhamahpuri Forest Division MH | 04/04/17 | Wild |
| 21 | Leopard | Beni | M | 06 Y | 65 kgs | Sehore Forest Division MP | 04/04/17 | Wild |

**Supplemenatary Table 2.** Number of raw paired-end reads generated in this study

| **Species** | **Sample ID** | **V3** | **ITS-1** | **Virome** | **Metagenome** |
| --- | --- | --- | --- | --- | --- |
| Lion | LN1 | 1018627 | 542379 | 2353670 | 5477522 |
| Lion | LN2 | 777915 | 479740 | 1990867 | 6080359 |
| Lion | LN3 | 1103048 | 289085 | 2995933 | 4809892 |
| Leopard | LP1 | 874816 | - | 1498280 | 3390288 |
| Leopard | LP2 | 1233008 | 988899 | 1350864 | 4836238 |
| Leopard | LP3 | 1667631 | 595497 | 1633481 | 6493043 |
| Leopard | LP4 | 1845223 | 699146 | 1813683 | 2415668 |
| Leopard | LP5 | 1104333 | 465683 | 1575738 | 4764210 |
| Leopard | LP6 | 1528473 | - | 1573469 | 6851851 |
| Leopard | LP7 | 1186200 | 393714 | 1591494 | 11701828 |
| Leopard | LP8 | 1810679 | 632200 | 1877317 | 5842294 |
| Leopard | LP9 | 1915951 | - | 2140368 | 3134851 |
| Tiger | TG1 | 1489161 | 1737284 | 1723545 | 4099469 |
| Tiger | TG2 | 1287143 | 941129 | 1139083 | 3578048 |
| Tiger | TG3 | 777769 | 938315 | 971666 | 5732572 |
| Tiger | TG4 | 1332560 | 783793 | 1136984 | 4327688 |
| Tiger | TG5 | 1309841 | - |  | 2711511 |
| Tiger | TG6 | 845590 | 1284591 |  | 2603324 |
| Tiger | TG7 | 884985 | - | 1284449 | 5963063 |
| Tiger | TG8 | 1792284 | 752464 | 1241308 | 8189020 |
| Tiger | TG9 | 1247675 | 667311 | 1554502 | 5144402 |

**Supplemenatary Table 3.** Correlations of bacterial and fungal taxa and fungal and viral taxa. Correlations with p-value < 0.05 are shown. No significant correlation was observed for bacterial and viral.

| **Correlations of bacterial and fungal taxa** | | | | |
| --- | --- | --- | --- | --- |
| **Bacterial_genera** | **Fungal_genera** | **test-statistic** | **estimate** | **p-value** |
| Collinsella | Fusarium | 1052 | -0.547 | 0.031 |
| [Ruminococcus] | Phoma | 1046 | -0.538 | 0.034 |
| [Ruminococcus] | Curvularia | 1018.249 | -0.497 | 0.050 |
| Sutterella | Aspergillus | 1120 | -0.647 | 0.008 |
| Sutterella | Chaetomium | 1108 | -0.629 | 0.011 |
|  |  |  |  |  |
| **Correlations of fungal and viral taxa** | | | | |
| **Fungal_genera** | **Viral_families** | **test-statistic** | **estimate** | **p-value** |
| Phoma | Caudovirales | 250 | 0.554 | 0.035 |
| Curvularia | Microviridae | 208.372 | 0.628 | 0.012 |
| Edenia | Microviridae | 247.925 | 0.557 | 0.031 |

**Supplementary Table 4.** Number of high quality metagenomic reads used for the construction of gene catalogue

| **SampleID** | **Species/Subspecies** | **Number of high quality reads** |  |
| --- | --- | --- | --- |
| LN1 | Lion | 9638076 | This study |
| LN2 | Lion | 10695733 | This study |
| LN3 | Lion | 8252589 | This study |
| LP1 | Indian leopard | 5856835 | This study |
| LP2 | Indian leopard | 8557826 | This study |
| LP3 | Indian leopard | 11311569 | This study |
| LP4 | Indian leopard | 4205110 | This study |
| LP5 | Indian leopard | 8210176 | This study |
| LP6 | Indian leopard | 11776653 | This study |
| LP7 | Indian leopard | 15331653 | This study |
| LP8 | Indian leopard | 10290938 | This study |
| LP9 | Indian leopard | 5396593 | This study |
| TG1 | Bengal tiger | 7123926 | This study |
| TG2 | Bengal tiger | 6214391 | This study |
| TG3 | Bengal tiger | 8194491 | This study |
| TG4 | Bengal tiger | 7183222 | This study |
| TG5 | Bengal tiger | 4713308 | This study |
| TG6 | Bengal tiger | 4585547 | This study |
| TG7 | Bengal tiger | 9810330 | This study |
| TG8 | Bengal tiger | 14750178 | This study |
| TG9 | Bengal tiger | 8991385 | This study |
| SRR6155883 | Amur tiger | 69141062 | (He et al., 2018) |
| SRR6155884 | Amur tiger | 63930742 | (He et al., 2018) |
| SRR6155885 | Amur tiger | 80324958 | (He et al., 2018) |
| SRR6256454 | Amur tiger | 90068341 | (He et al., 2018) |
| SRR6256455 | Amur tiger | 44149168 | (He et al., 2018) |
| SRR6256456 | Amur tiger | 24248278 | (He et al., 2018) |

**Supplementary Table 5.** The top 10 most abundant CAZy subfamilies observed in the four *Panthera* phylotypes

| **CAZy** | **Lion** | **Leopard** | **Bengal tiger** | **Amur tiger** |
| --- | --- | --- | --- | --- |
| GT2 | 0.162 | 0.135 | 0.149 | 0.169 |
| GT4 | 0.104 | 0.079 | 0.100 | 0.114 |
| CBM50 | 0.052 | 0.057 | 0.064 | 0.050 |
| GH23 | 0.046 | 0.059 | 0.032 | 0.027 |
| GH13 | 0.037 | 0.030 | 0.030 | 0.035 |
| GH73 | 0.029 | 0.069 | 0.041 | 0.015 |
| GH24 | 0.024 | 0.048 | 0.051 | 0.007 |
| GT51 | 0.019 | 0.014 | 0.017 | 0.021 |
| GH1 | 0.016 | 0.012 | 0.017 | 0.022 |
| GH2 | 0.015 | 0.012 | 0.016 | 0.016 |
| GH20 | 0.015 | 0.016 | 0.020 | 0.005 |
| GH3 | 0.014 | 0.011 | 0.015 | 0.015 |

**Supplementary Table 6.** The top 10 most abundant MEROPS subfamilies observed in the four *Panthera* phylotypes

| **Category** | **Lion** | **Leopard** | **Bengal tiger** | **Amur tiger** | **Description** |
| --- | --- | --- | --- | --- | --- |
| Family I39 unassigned peptidase inhibitors | 0.043 | 0.054 | 0.038 | 0.044 |  |
| Family M38 non-peptidase homologues | 0.044 | 0.024 | 0.032 | 0.037 |  |
| Subfamily M23B unassigned peptidases | 0.059 | 0.136 | 0.085 | 0.037 | Zinc metallopeptidases. These are Gly-Gly endopeptidases. |
| Family U69 unassigned peptidases | 0.005 | 0.006 | 0.013 | 0.029 | AIDA-I self-cleaving autotransporter protein |
| Subfamily M23B non-peptidase homologues | 0.014 | 0.017 | 0.024 | 0.021 |  |
| Family C26 unassigned peptidases | 0.013 | 0.011 | 0.014 | 0.019 | These peptidases have gamma-glutamyl hydrolase activity. Cystiene peptidases |
| Family C26 non-peptidase homologues | 0.018 | 0.011 | 0.015 | 0.019 |  |
| Family U32 unassigned peptidases | 0.013 | 0.011 | 0.014 | 0.016 | collagenase |
| Family S33 unassigned peptidases | 0.011 | 0.007 | 0.017 | 0.016 | serine peptidase. They include prolinases (Pro-Xaa dipeptidase, 3.4.13.18), prolyl aminopeptidases (3.4.11.5), and L-amino acid amidases. |
| Family I87 unassigned peptidase inhibitors | 0.016 | 0.016 | 0.013 | 0.015 |  |
| Family S9 unassigned peptidases | 0.011 | 0.009 | 0.015 | 0.014 | serine peptidases |
| Family M20D unassigned peptidases | 0.018 | 0.008 | 0.009 | 0.010 | The enzymes are carboxydipeptidases and primarily specific for Met-X dipeptides. |
| Family C40 unassigned peptidases | 0.037 | 0.037 | 0.048 | 0.009 | dipeptidyl-peptidase VI |
| Family S11 unassigned peptidases | 0.014 | 0.008 | 0.014 | 0.008 | D-Ala-D-Ala carboxypeptidase A |
| Subfamily M20A unassigned peptidases | 0.013 | 0.006 | 0.007 | 0.007 | glutamate carboxypeptidase |
| Family C40 non-peptidase homologues | 0.004 | 0.029 | 0.003 | 0.004 |  |
| Family S14 non-peptidase homologues | 0.005 | 0.015 | 0.005 | 0.003 |  |
| Subfamily S26B unassigned peptidases | 0.002 | 0.038 | 0.001 | 0.001 | signalase 21 kDa component |
| Family N9 unassigned peptide lyases | 0.011 | 0.048 | 0.003 | 0.000 | intein-containing V-type proton ATPase catalytic subunit A |

**Supplementary Table 7.** The sample information of 62 species used in the PCA analysis of 16S rRNA bacterial amplicons

| **SampleID** | **Species** | **Animal Name** | **Gender** | **place** | **Location** | **Number of reads** | **Reads Type** |
| --- | --- | --- | --- | --- | --- | --- | --- |
| LN1-A | Lion | Shiva | M | Rescue_circus | India | 1018627 | Paired |
| LN2-A | Lion | Shivani | F | Rescue_circus | India | 777915 | Paired |
| LN3-A | Lion | Jamuna II | F | Rescue_circus | India | 1103048 | Paired |
| LP1-A | Indian leopard | Puri | F | Wild | India | 874816 | Paired |
| LP2-A | Indian leopard | Panna | M | Wild | India | 1233008 | Paired |
| LP3-A | Indian leopard | Jabalpur | M | Wild | India | 1667631 | Paired |
| LP4-A | Indian leopard | Jobat | M | Wild | India | 1845223 | Paired |
| LP5-A | Indian leopard | Sheru | M | Wild | India | 1104333 | Paired |
| LP6-A | Indian leopard | Nena | F | Wild | India | 1528473 | Paired |
| LP7-A | Indian leopard | Nimu | M | Wild | India | 1186200 | Paired |
| LP8-A | Indian leopard | Raju | M | Wild | India | 1810679 | Paired |
| LP9-A | Indian leopard | Beni | M | Wild | India | 1915951 | Paired |
| TG1-A | Bengal tiger | Kanha | M | Wild | India | 1489161 | Paired |
| TG2-A | Bengal tiger | Panna | M | Wild | India | 1287143 | Paired |
| TG3-A | Bengal tiger | Bandhu | M | Wild | India | 777769 | Paired |
| TG4-A | Bengal tiger | Gauri | F | Captive | India | 1332560 | Paired |
| TG5-A | Bengal tiger | Matakkali | F | Wild | India | 1309841 | Paired |
| TG6-A | Bengal tiger | Riddhi (White tiger) | F | Captive | India | 845590 | Paired |
| TG7-A | Bengal tiger | Priya | F | Rescue_circus | India | 884985 | Paired |
| TG8-A | Bengal tiger | Kamlesh | F | Captive | India | 1792284 | Paired |
| TG9-A | Bengal tiger | Ganga | F | Captive | India | 1247675 | Paired |
| ERR1804913 | Fox | Fox1 |  |  | China | 102207 | Paired |
| ERR1804914 | Fox | Fox2 |  |  | China | 115843 | Paired |
| ERR1804915 | Fox | Fox3 |  |  | China | 106380 | Paired |
| ERR1804916 | Fox | Fox4 |  |  | China | 107746 | Paired |
| ERR1804917 | Fox | Fox5 |  |  | China | 190488 | Paired |
| ERR2821250 | Wolf | 10936.FD.0099.wolf | F |  | France | 152687 | Single |
| ERR2821251 | Wolf | Wolf | M |  | France | 79355 | Single |
| ERR2821256 | Cheetah | 10936.FD.0168.cheetah | F |  | France | 189463 | Single |
| ERR2821271 | Wild dog | 10936.FD.0268.wilddog | M |  | France | 126820 | Single |
| ERR2821276 | Wolf | Iberian wolf |  | Captive | France | 144654 | Single |
| ERR2821310 | Cheetah | qiita_sid_10936:10936.TS.031.cheetah | | wild | South Africa | 69505 | Single |
| ERR2821311 | Cheetah | qiita_sid_10936:10936.TS.032.cheetah | | wild | South Africa | 134679 | Single |
| ERR2821340 | Wild dog | 10936.TS.207.wilddog |  | wild | South Africa | 140555 | Single |
| ERR2821341 | Wild dog | qiita_sid_10936:10936.TS.208.wilddog | | wild | South Africa | 191116 | Single |
| ERR2821342 | Wild dog | 10936.TS.209.wilddog |  | wild | South Africa | 81740 | Single |
| ERR2821343 | Wild dog | 10936.TS.210.wilddog |  | wild | South Africa | 120527 | Single |
| ERR2821345 | Wolf | zurich.wolf.1 |  | captive | Switzerland | 119714 | Single |
| SRR6147361 | Malayan tiger | tiger2 |  |  | USA | 22009 | Single |
| SRR6147362 | Malayan tiger | tiger1 |  |  | USA | 78332 | Single |
| SRR6841704 | Caracal caracal | CC |  |  | China: Jinan | 1458741 | Single |
| SRR7229827 | North Chinese leopard | J2 |  |  | China: Shanxi | 83512 | Single |
| SRR7229828 | Amur leopard | O6 |  |  | China: Jilin | 52292 | Single |
| SRR7229829 | North Chinese leopard | J10 |  |  | China: Shanxi | 80658 | Single |
| SRR7229830 | North Chinese leopard | J9 |  |  | China: Shanxi | 62895 | Single |
| SRR7229831 | Amur leopard | O7 |  |  | China: Jilin | 50716 | Single |
| SRR7229832 | North Chinese leopard | J1 |  |  | China: Shanxi | 81656 | Single |
| SRR7229833 | Amur leopard | O8 |  |  | China: Jilin | 86540 | Single |
| SRR7229834 | North Chinese leopard | J12 |  |  | China: Shanxi | 51784 | Single |
| SRR7229835 | North Chinese leopard | J11 |  |  | China: Shanxi | 64069 | Single |
| SRR7229836 | Amur leopard | O1 |  |  | China: Jilin | 60144 | Single |
| SRR7229837 | North Chinese leopard | J13 |  |  | China: Shanxi | 88896 | Single |
| SRR7229838 | Amur leopard | O3 |  |  | China: Jilin | 57864 | Single |
| SRR7229839 | Amur leopard | O2 |  |  | China: Jilin | 72103 | Single |
| SRR7229840 | Amur leopard | O5 |  |  | China: Jilin | 85225 | Single |
| SRR7229841 | Amur leopard | O4 |  |  | China: Jilin | 67612 | Single |
| SRR7229842 | North Chinese leopard | J7 |  |  | China: Shanxi | 91079 | Single |
| SRR7229843 | North Chinese leopard | J4 |  |  | China: Shanxi | 74305 | Single |
| SRR7229844 | North Chinese leopard | J8 |  |  | China: Shanxi | 87327 | Single |
| SRR7229845 | North Chinese leopard | J6 |  |  | China: Shanxi | 81686 | Single |
| SRR7229846 | North Chinese leopard | J3 |  |  | China: Shanxi | 70013 | Single |
| SRR7229847 | North Chinese leopard | J5 |  |  | China: Shanxi | 63857 | Single |

**Supplementary Figures**


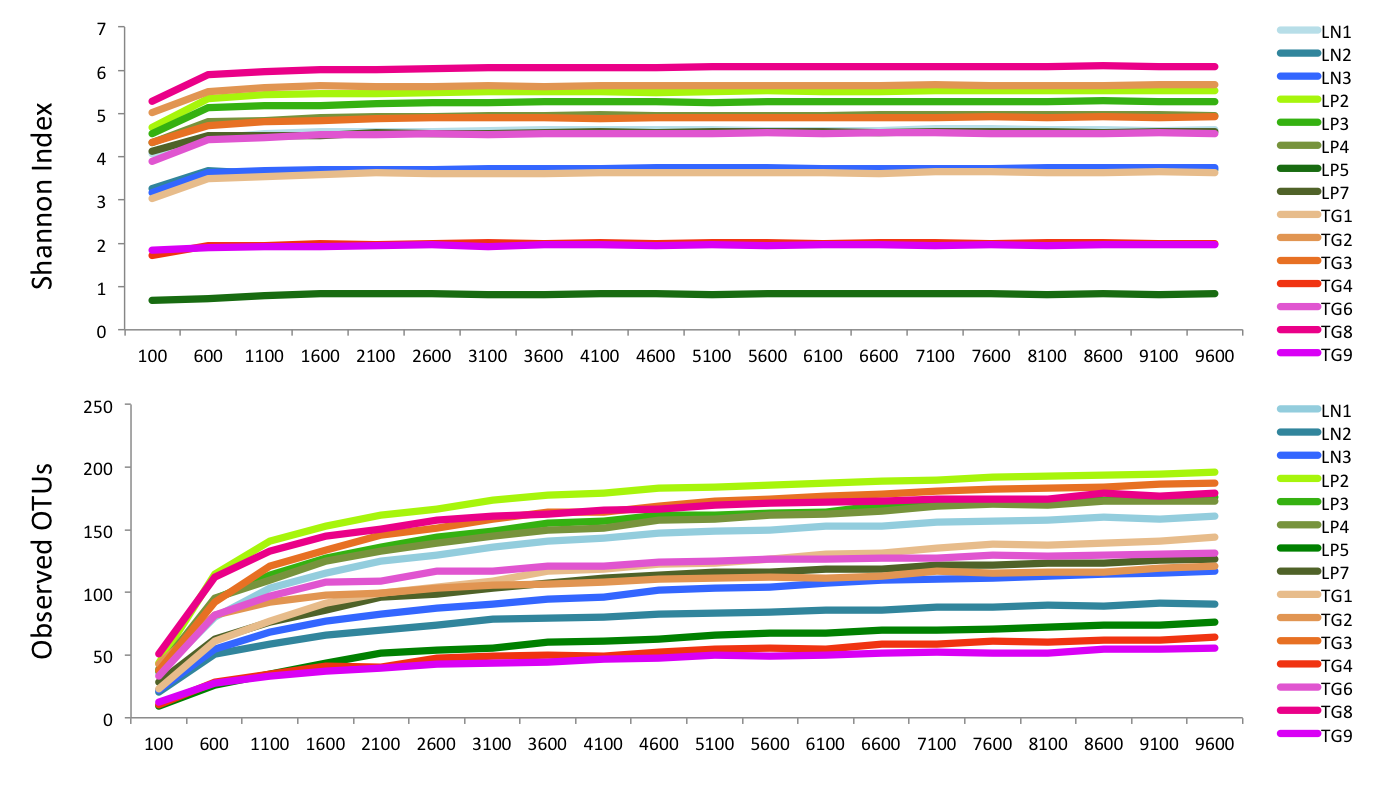


**Supplementary Figure 1.** Rarefaction curves showing Shannon diversity indicies and number of observed OTUs from the ITS1 amplicon sequences.


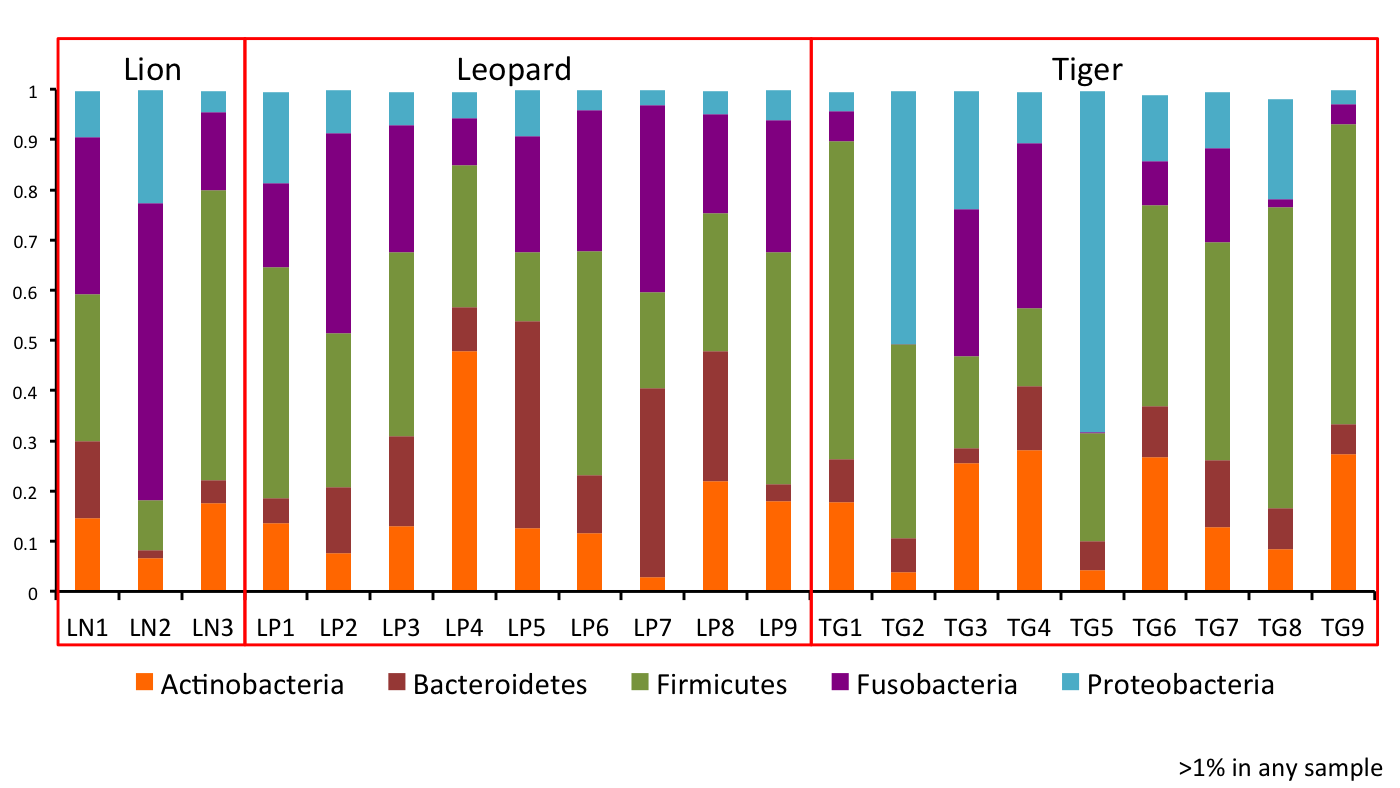


**Supplementary Figure 2**. The phylum level distribution of bacteria present in the *Panthera* gut.

**
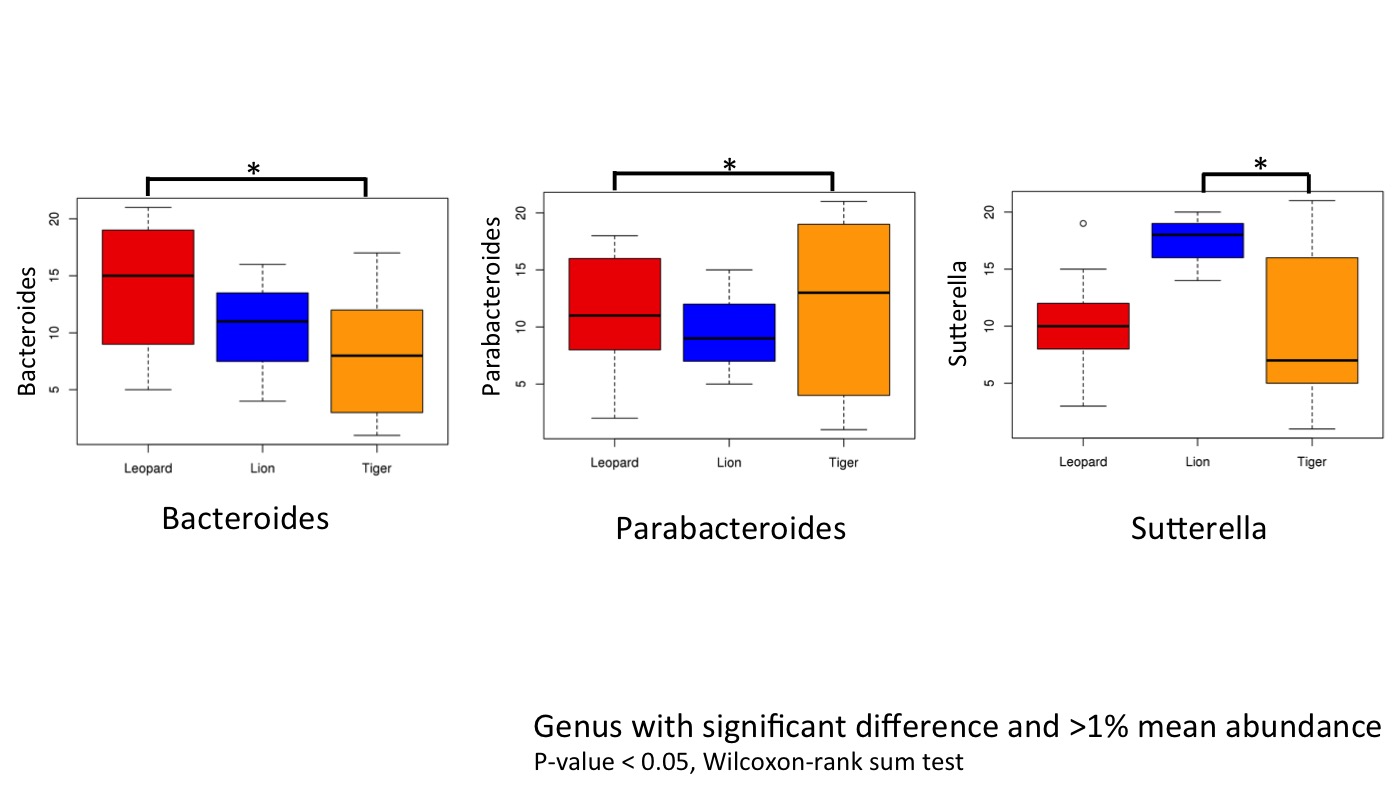
**

**Supplementary Figure 3.** Significant differences observed in the bacterial genus abundances.

**
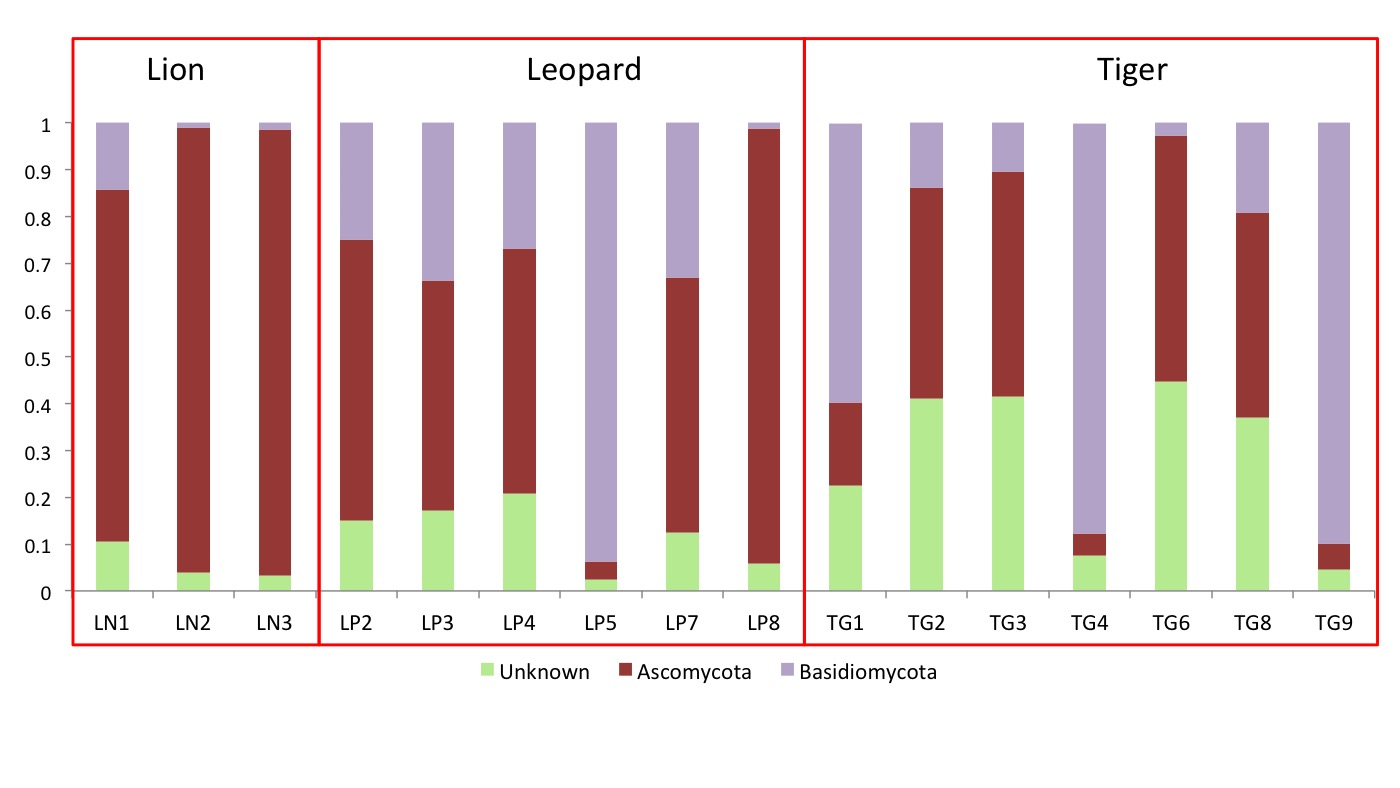
**

**Supplementary Figure 4**. The phylum level distribution of fungi present in the *Panthera* gut.

**Supplementary Text**

The fecal samples of 21 individuals of *Panthera* species were collected from Van Vihar, Bhopal, India. These samples included nine Bengal tigers, nine Indian leopards and three lions (Asian-African hybrid) (**Supplementary** **Table 1**). None of the individual was suffering from any disease and their health was monitered at Van Vihar, Bhopal on a regular basis. The regular diet of these individuals included raw buffalo meat once a day with one day (Friday) fasting in a week. The quantity of the food varied based on the species, sex and size of the individual. These individuals consumed approximately 7-8 L of water each day. Supplements such as calcium and vitamins were also provided to these individuals from time to time in the form of tablets. The fecal samples of these individuals were collected in April 2017.

**References**

He F, Liu D, Zhang L, Zhai J, Ma Y, Xu Y*, et al.* (2018). Metagenomic analysis of captive Amur tiger faecal microbiome. BMC Vet Res 14(1), 379. doi: 10.1186/s12917-018-1696-5.
